# Effect of Gender on Coagulation Functions: A Study in Metastatic Colorectal Cancer Patients Treated with Bevacizumab, Irinotecan, 5-Fluorouracil, and Leucovorin

**DOI:** 10.1155/2014/473482

**Published:** 2014-08-21

**Authors:** Cemil Bilir, Hüseyin Engin, Yasemin Bakkal Temi

**Affiliations:** Bülent Ecevit University School of Medicine, Department of Internal Medicine, Division of Medical Oncology, 67100 Zonguldak, Turkey

## Abstract

*Introduction.* We designed this study to evaluate how coagulation parameters are changed in metastatic colorectal cancer (mCRC) patients treated with bevacizumab, irinotecan, 5-fluorouracil, and leucovorin (FOLFIRI). *Methods.* A total of 48 mCRC patients who initially received bevacizumab with FOLFIRI were eligible for this study. Thirty-four patients were analyzed at baseline and on the 4th, 8th, and 12th cycles of chemotherapy. *Results.* There were 19 male and 15 female patients. Baseline characteristics of the groups were similar, but women had better overall survival than men (14 months versus 12 months, *P* = 0.044). D-dimer levels decreased significantly after the 12th cycle compared with baseline in men but not in women. Men and women had increased levels of serum fibrinogen at the early cycles, but these increased fibrinogen levels continued after the 4th cycle of chemotherapy only in women. In addition, serum fibrinogen levels did not significantly change, but aPTT levels decreased in men. *Discussion.* The major finding of this study is that bevacizumab-FOLFIRI chemotherapy does not promote changes in the coagulation system. If chemotherapy treatment and the possible side effects of FOLFIRI-bevacizumab treatment are well managed, then alterations of the coagulation cascade will not have an impact on overall survival and mortality.

## 1. Introduction

Bevacizumab is typically used in combination with fluoropyrimidine-based chemotherapy for the treatment of patients with metastatic colorectal cancer (mCRC) [1]. In a pivotal phase III trial of first-line mCRC, bevacizumab in combination with standard irinotecan/fluorouracil (FOLFIRI) chemotherapy increased tumor response rate by 10% and significantly lengthened the overall survival [1, 2]. In the bevacizumab arm, the patients had an increased incidence of thrombotic events compared with the control arm (19.4% versus 16.2%, resp.), but this result was not significant [1]. In a meta-analysis, a total of 7956 patients diagnosed with many types of solid tumors from 15 randomized trials were identified. The authors revealed that patients treated with bevacizumab had a statistically significant increased risk of venous thromboembolic events (VTEs), with an RR of 1.33 (95% CI, 1.13–1.56, *P* < 0.001) compared with the controls [3]. Hurwitz et al. reported a meta-analysis of 6,055 patients in 10 randomized studies [4]. This study concluded that the addition of bevacizumab to chemotherapy did not significantly increase the risk of VTEs versus chemotherapy alone. The risk for VTEs was attributed to tumor and host factors [4]. Epistaxis, hemoptysis, hematemesis, gastrointestinal bleeding, vaginal bleeding, and brain hemorrhage have been reported as hemorrhagic toxicities associated with bevacizumab [5]. These controversial findings open the possibility that factors other than bevacizumab, such as drugs, cancer, age, gender, or comorbidities, can cause VTEs. Therefore, we designed this study to evaluate how coagulation parameters, such as the international normalized ratio (INR), activated partial thromboplastin time (aPTT), and fibrinogen and D-dimer levels, can be altered in mCRC patients treated with FOLFIRI-bevacizumab chemotherapy.

## 2. Materials and Methods

A total of 48 metastatic colorectal cancer patients who had initially received bevacizumab combined with the FOLFIRI regimen were eligible for the study. The study was conducted in the Medical Oncology Clinic of the Bulent Ecevit University School of Medicine between the period of May 2010 and May 2013. Patients with a previous history of hematological disease, those who have been taking anticoagulant therapy, and those with chronic disease, such as liver cirrhosis or renal failure, were excluded from the study. During the bevacizumab treatment period, all patients who had Grade 3-4 bleeding were excluded from the study. Written and verbal consents were obtained from all the patients for chemotherapy and study enrollment. Treatment responses were measured between every 2 and 3 months. If a patient progressed or discontinued bevacizumab for any reason (e.g., bleeding, intestinal perforation, and metastasectomy), he or she was excluded from the study. At the end of the study, a total of 34 patients were included and analyzed.

### 2.1. Drug Administration

All patients received the following treatment regimen: bevacizumab 5 mg/kg IV combined with irinotecan 180 mg/m^2^ IV (Day 1), leucovorin 400 mg/m^2^ IV (Day 1), and 5-fluorouracil (400 mg/m^2^ IV bolus and then 2400 mg/m^2^ continuous infusion 46 h) IV, once every 2 weeks for 12 cycles. All measurements were repeated at baseline and on the 4th, 8th, and 12th cycles of chemotherapy. Chemotherapy was continued beyond the 12th cycle for patients who had a partial response or stable disease.

### 2.2. Sample Collection

Blood samples were collected before drug administration at baseline and on Day 1 of chemotherapy of the 4th, 8th, and 12th cycles using a 19-gauge needle under minimum stasis. Platelet counts were analyzed using a Beckman Coulter Gen-S (SM, USA) automated blood counting device.

The conventional coagulation parameters, such as the international normalized ratio (INR), activated partial thromboplastin time (aPTT), fibrinogen, and D-dimer, were measured using a fully automated STA compact device from Diagnostica Stago.

### 2.3. Statistical Analysis

Treatment outcomes were estimated as response rate (RR), disease control rate, OS, and progression-free survival (PFS). OS was defined as the time between the date of metastatic disease diagnosis and the date of death from any cause. PFS was defined as the time from the date of metastatic disease diagnosis to the date of disease progression or death from any cause.

Overall survival was calculated using the Kaplan-Meier method and the log-rank test. *P* < 0.05 was considered to be significant. The appropriateness of data to normal ranges was controlled using the Shapiro-Wilk test. Data were analyzed using the two-way ANOVA and nonparametric tests. Data in the tables are presented as the mean and standard error (SE) or median and interquartile range (IR) of data. A probability value of less than 0.05 was considered to be statistically significant.

## 3. Results

In this study, there were 19 male and 15 female patients diagnosed with mCRC. The mean age of women was 60.1 (±7) and that of men was 61.5 (±9.9); there was no significant difference between the groups (*P* = 0.59). Baseline characteristics of the groups are shown in Table 1. All parameters, except OS, had no statistical significance. Women had greater OS than men. Men had more liver and lung metastases compared with women, but the difference was not significant (15 versus 9, *P* = 0.7).

**Figure 1:**
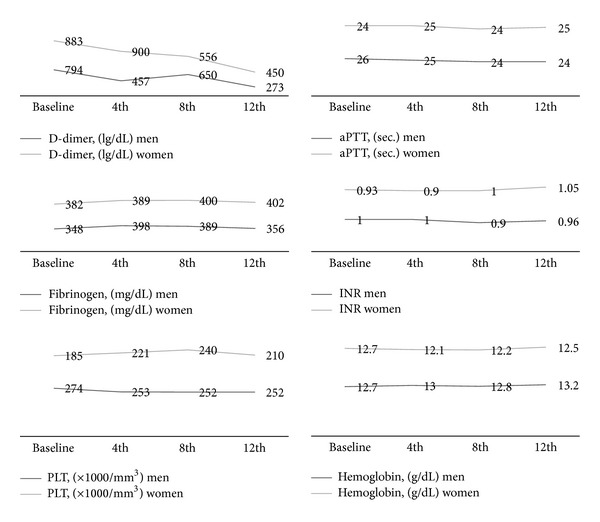
Results of laboratory parameters at baseline and on Day 1 of cycles 4, 8, and 12.

Results of laboratory parameters at baseline and on Day 1 of the 4th, 8th, and 12th cycles are presented in Figure 1. Delta values were defined as the differences between the baseline and each of the following cycles: 4th, 8th, and 12th. D-dimer levels decreased significantly after the 12th cycle compared with the baseline in men but not in women. Both men and women have increased levels of serum fibrinogen at the early cycles, but increased fibrinogen levels continued after the 4th cycle of chemotherapy only in women. Conversely, fibrinogen levels and serum aPTT levels decreased in men but not in women. Serum INR levels did not change significantly in the first eight cycles of chemotherapy in both men and women. However, after the 12th cycle, INR levels increased significantly in women. This increase was statistically significant but without clinical meaning. Women have a higher risk of anemia than men in the early stages of treatment because their Hb levels decreased from 12.7 to 12.1, whereas there was no decrease detected in men. Cox regression analysis did not find any correlation of laboratory parameters with OS and PFS.

Some coagulation parameters have differences according to metastatic sites. In women, baseline fibrinogen levels were higher in liver metastasis compared with other metastatic sites, such as bone, peritoneum, and lung (381 mg/dL versus 348 mg/dL, *P* = 0.007). Additionally, serum baseline PLT levels were lower in liver metastasis compared with the other sites (170,000/mm^3^ versus 262,000/mm^3^, *P* = 0.007). With regard to changes in the parameters because of chemotherapy, Hb levels were different in the groups according to the metastatic site. There was a significant decrease in the Hb levels in the early period of chemotherapy especially in women. Dropping Hb levels were more prominent in peritoneal and bone metastases compared with liver metastasis (0.66 gr/dL versus 0.35 gr/dL, *P* = 0.04). There was no correlation between the laboratory parameters and metastatic sites in men. After the study termination, 1 man and 1 woman (6% of the study population) had deep vein thrombosis without pulmonary embolism, and there also was no Grade 3-4 bleeding.

## 4. Discussion

In this study, we found that FOLFIRI-bevacizumab chemotherapy has some changes on coagulation parameters such as decreased D-dimer levels in men, increased fibrinogen levels in both men and women in the early stage of treatment, and increased INR in women. Despite these changes, none of these variables had an effect on OS and/or PFS.

A few small studies have reported changes in coagulation system markers in response to breast cancer chemotherapy, and these reports supported the development of a chemotherapy-induced hypercoagulable state [6]. Kirwan et al. also reported a well-designed prospective study on the markers of hemostasis (thrombin-antithrombin (TAT), fibrinogen, D-dimer, and platelet count) and functional clotting assays (prothrombin time (PT) and aPTT and procoagulants tissue factor (TF), cancer procoagulant (CP), and plasma vascular endothelial growth factor (pVEGF)), which were measured before chemotherapy and at 24 h, 4 days, 8 days, and 3 months after chemotherapy in patients with breast cancer. The authors revealed that the coagulation markers and procoagulants were increased before chemotherapy in patients who subsequently developed VTE [7]. Additionally, the authors revealed that aPTT showed a marked decrease within 24 h of chemotherapy; however, it was more pronounced in patients who subsequently developed VTE. The reduction in aPTT was maintained for up to 3 months [7], whereas a marked prolongation of PT was detected at 8 days and occurred only in patients who subsequently developed VTE [7]. In a lung cancer study, patients receiving chemotherapy revealed an early reduction of aPTT (at Days 2, 5, and 7 after treatment) and a slightly later decrease of PT (at Days 5, 7, and 14 after treatment) [8]. According to these findings, we speculate that there have been some alterations in the coagulation systems in patients who received chemotherapy. In colorectal cancer, there is only one small study published by Ustuner et al. They examined changes in coagulation parameters in 18 metastatic colorectal cancer patients and did not find any significant changes in platelet count, PT, and aPTT at baseline and in the subsequent chemotherapy cycles [5]; however, their study population was small and had no gender differentiation [5]. In our study, we found a shortened aPTT after the 8th cycle in men but not in women. However, this decrease remains within normal limits, and there was no clinical finding, such as VTE, in any patients for the duration of our study. Additionally, similar to previous breast cancer studies, we found minimal INR prolonged from the 0th cycle of the chemotherapy to the 12th cycle in women; only two women had Grade 1-2 bleeding during the study, and INR prolongation was detected after the 12th cycle. We may have been able to show more INR prolongation if we followed the patients beyond the 12th cycle, but we did not have prolonged follow-up results.

In this study, women had greater OS compared with men, and this finding may be a reason for men to have more liver and lung metastases compared with women, so men had a higher metastatic tumor burden.

In patients with cancer, the major causes are direct myelotoxicity from chemotherapeutic drugs and cytokine-mediated inhibition of erythropoiesis [9]. We showed a drop in Hb after the 4th cycle of FOLFIRI-bevacizumab chemotherapy in women but not in men. After the 4th cycle, we did not find any significant change in both men and women, but we must consider that, during the course of treatment, especially after the 4th to 6th cycles, some patients received blood transfusions.

Ustuner et al. did not find any significant changes in D-dimer and fibrinogen levels after the FOLFIRI-bevacizumab treatment [5], but we found a nonsignificant reduction in D-dimer levels in women and a significant decrease after the 12th cycle in men. This result is interesting because it reveals that D-dimer has a prognostic value in patients with colorectal and lung cancers [10]. In addition, Altiay et al. reported a significant correlation between response to chemotherapy and D-dimer levels in patients with both local and advanced lung cancer [11]. In our study, we showed a significant decrease in D-dimer levels in men; this reason may be the cause of higher metastatic tumor burden in men, and thus, after cancer chemotherapy, tumor burden and D-dimer can decrease; however, there was no significant correlation with overall survival. We also found a significant increase in fibrinogen levels in women, but there was no significant correlation with overall survival.

The major limitation of our study was having limited number of patients. We have only 34 patients with colorectal cancer, and we could not show the long-term outcomes such as VTE and/or bleeding complications. Only two patients had deep vein thrombosis after the chemotherapy; thus, a limited number of these patients avoid the statistical analyses. To our knowledge, there are some reports similar to our study that revealed the changes of coagulation parameters with cancer chemotherapy, but our study initially reported that these changes did not have any negative impact on OS in patients with colorectal cancer.

In conclusion, FOLFIRI-bevacizumab chemotherapy altered the coagulation functions according to gender. Although FOLFIRI-bevacizumab chemotherapy has some effects, these changes do not have any negative impact on overall survival and progression-free survival. If chemotherapy treatment and the possible side effects of FOLFIRI-bevacizumab are well managed, then alterations of the coagulation cascade will not have any impact on overall survival and mortality.

## Figures and Tables

**Table 1:** Baseline characteristics of the groups.

	Women (minimum–maximum) *n* = 15	Men (minimum–maximum) *n* = 19	*P* value
Age, years	60	61.5	0.85
Colon cancer	12	14	—
Rectal cancer	3	5	—
Adenocarcinoma	15	19	
Grades 1-2	5	4	
Grade 3	10	15	0.4
Metastases site			
Liver	8	12	
Lung	1	3	
Bone	3	4	0.8
Diabetes	3	4	
Hypertension	2	5	1
D-dimer (mg/dL)	883 (106–2233)	794 (206–2760)	0.88
Fibrinogen (mg/dL)	382 (330–415)	348 (250–630)	0.73
aPTT (sec)	24 (19–29)	26 (19–29)	0.26
INR	0.93 (0.8–1.06)	1.0 (0.8–1.1)	0.44
Platelet count (×1000 mm^3^)	185 (160–274)	274 (165–468)	0.057
Hemoglobin, gr/dL	12.7 (11.9–13.3)	12.7 (10.7–16)	0.98
Creatinine, mg/dL	0.9 (0.7–1.1)	0.8 (0.5–1.1)	0.17
White blood cell, mm^3^	5600 (4400–13500)	6600 (3900–11000)	0.46
PFS, months	8 (6–11)	9 (4–14)	0.7
OS, months	14 (10–33)	12 (6–28)	0.044

aPTT: activated partial thromboplastin time, INR: international normalized ratio, PFS: progression-free survival, and OS: overall survival.

## References

[1] Hurwitz H, Fehrenbacher L, Novotny W (2004). Bevacizumab plus irinotecan, fluorouracil, and leucovorin for metastatic colorectal cancer. *The New England Journal of Medicine*.

[2] Ferroni P, Formica V, Roselli M, Guadagni F (2010). Thromboembolic events in patients treated with anti-angiogenic drugs. *Current Vascular Pharmacology*.

[3] Nalluri SR, Chu D, Keresztes R, Zhu X, Wu S (2008). Risk of venous thromboembolism with the angiogenesis inhibitor bevacizumab in cancer patients: a meta-analysis. *JAMA—Journal of the American Medical Association*.

[4] Hurwitz HI, Saltz LB, van Cutsem E (2011). Venous thromboembolic events with chemotherapy plus bevacizumab: a pooled analysis of patients in randomized phase II and III studies. *Journal of Clinical Oncology*.

[5] Ustuner Z, Akay OM, Keskin M, Kuş E, Bal C, Gulbas Z (2012). Evaluating coagulation disorders in the use of bevacizumab for metastatic colorectal cancer by thrombelastography. *Medical Oncology*.

[6] Pectasides D, Tsavdaridis D, Aggouridaki C (1999). Effects on blood coagulation of adjuvant CNF (cyclophosphamide, novantrone, 5-fluorouracil) chemotherapy in stage II breast cancer patients. *Anticancer Research*.

[7] Kirwan CC, McDowell G, McCollum CN, Kumar S, Byrne GJ (2008). Early changes in the haemostatic and procoagulant systems after chemotherapy for breast cancer. *British Journal of Cancer*.

[8] Gabazza EC, Taguchi O, Yamakami T (1992). Coagulation-fibrinolysis system and markers of collagen metabolism in lung cancer. *Cancer*.

[9] Spivak JL (2005). The anaemia of cancer: death by a thousand cuts. *Nature Reviews Cancer*.

[10] Komurcuoglu B, Ulusoy S, Gayaf M, Guler A, Ozden E (2011). Prognostic value of plasma D-dimer levels in lung carcinoma. *Tumori*.

[11] Altiay G, Ciftci A, Demir M (2007). High plasma d-dimer level is associated with decreased survival in patients with lung cancer. *Clinical Oncology*.

